# Tannic Acid-Induced Surface-Catalyzed Secondary Nucleation during the Amyloid Fibrillation of Hen Egg-White Lysozyme

**DOI:** 10.3390/ijms19124009

**Published:** 2018-12-12

**Authors:** Jing Tian, Yang Yu, Yao Wang, Haoyi Li, Lujuan Yang, Baoan Du, Gang Ma

**Affiliations:** Key Laboratory of Medicinal Chemistry and Molecular Diagnosis of Ministry of Education, Key Laboratory of Analytical Science and Technology of Hebei Province, College of Chemistry and Environmental Science, Hebei University, Baoding 071002, China; tianjing_ir@outlook.com (J.T.); yuyang_ir@outlook.com (Y.Y.); wangyao_ir@outlook.com (Y.W.); haoyili.zh@gmail.com (H.L.); yanglujuan_ir@outlook.com (L.Y.)

**Keywords:** amyloid, lysozyme, tannic acid, fibrillation, atomic force microscopy, fluorescence spectroscopy

## Abstract

Amyloid fibrillation by hen egg white lysozyme under the influence of tannic acid was investigated by atomic force microscopy and fluorescence spectroscopy. Tannic acid was found to be able to induce the formation of amyloid fibrils with an interesting mixed morphology. Such morphology features with the existence of areas of thickening alternating with areas of normal height. This novel modulation effect of tannic acid on amyloid fibrillation was interpreted by the established surface-catalyzed secondary nucleation theory. We further performed a fluorescence quenching study to investigate the intermolecular interaction between tannic acid and lysozyme. The results support that lysozyme and tannic acid interact with each other mainly through hydrophobic interactions. We also discussed why hydrogen-bonding interaction is not a dominant factor in the interaction between tannic acid and lysozyme though tannic acid contains a significant amount of hydroxyl groups. Our work provides new insight into the effect of tannic acid, a well-known amyloid inhibitor, on amyloid fibrillation.

## 1. Introduction

Amyloid fibrillation is a protein and peptide self-assembling process through which proteins and peptides form insoluble fibril-like aggregates known as amyloid fibrils [1]. The basic structural unit of an amyloid fibril consists of two interdigitatedly paired β-sheets, and such a paired β-sheet structure displays a unique cross-β-type pattern under X-ray diffraction [2,3]. The width of an amyloid fibril is at the nanoscale and its length can reach several microns. Therefore, amyloid fibril can be considered a protein or peptide-based nanowire. Amyloid fibrils can be stained by specific dyes, such as thioflavin T (ThT) and Congo red [4,5,6]. The presence of amyloid fibrils in tissue and organs in the human body is the pathological hallmark of about 40 neurodegenerative, systemic, and nonsystemic diseases, including Alzheimer’s, Parkinson’s, and prion disease, and type II diabetes [7]. In addition, amyloid fibrils have attracted much attention among material scientists owing to their excellent mechanical properties and good biocompatibility [8,9,10]. In fact, amyloid fibrils have been used as building blocks in a variety of novel nanomaterials in recent years and have shown great promise in a wide range of applications, including sensing, drug delivery, and tissue engineering [9,11,12].

Amyloid fibrillation is a rather complex process, as several nucleation mechanisms could coexist during the course of fibrillation [13]. Two nucleation mechanisms have been proposed so far: the primary nucleation mechanism and the secondary nucleation mechanism. In addition, there are two different types of secondary nucleation mechanisms, namely, nucleation by fragmentation and surface-catalyzed nucleation. In the primary nucleation mechanism, new aggregates are formed through interactions solely between soluble monomers; in nucleation by fragmentation, new aggregates are generated through fibril fragmentation that would generate more new ends to allow further growth of the fibril. In both mechanisms, fibril elongation occurs through the addition of monomers at the ends of the fibril. In surface-catalyzed nucleation, new aggregates are generated on the surface of preformed fibril. Intuitively, with this mechanism, monomers use the surface of a preformed fibril as a type of template to nucleate [13,14,15,16,17].

**Figure 1:**
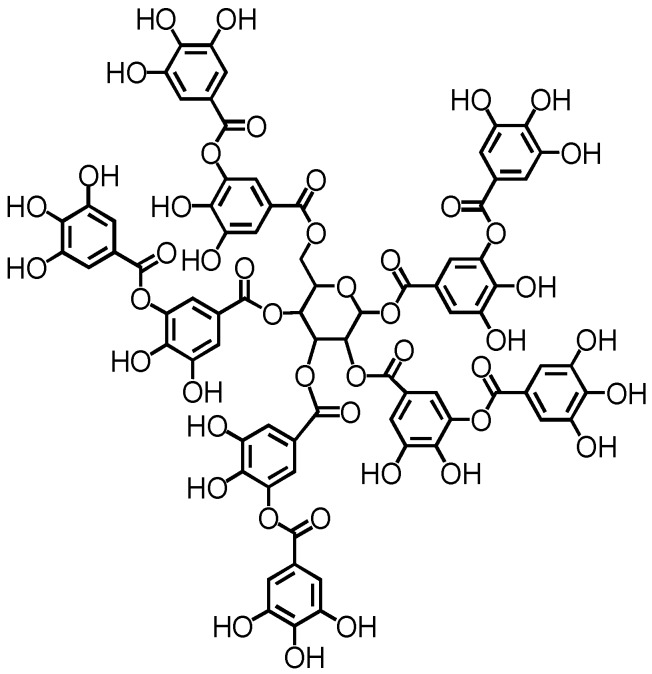
Molecular structure of tannic acid.

Understanding the factors that could modulate amyloid fibrillation is no doubt fundamentally important in amyloid research. It could not only lead to the discovery of novel amyloid inhibitors that could be used to treat amyloid-related diseases, but also lead to the development of amyloid-based materials with novel morphologies and properties. In this work, we aim to investigate the modulation effect of tannic acid on amyloid fibrillation using hen egg white lysozyme as a model amyloid system. Hen egg white lysozyme is a widely used model system in amyloid research [18,19,20,21,22,23,24,25,26,27,28,29,30,31,32,33,34]. Tannic acid is a type of polyphenol, as shown in Figure 1, and many polyphenol compounds including tannic acid have been found to possess amyloid-inhibition capabilities [35]. As for tannic acid, Yamada and coworkers found that tannic acid could not only inhibit the amyloid fibrillation of Aβ(1–40), Aβ(1–42), and α-synuclein, but also destabilize the preformed amyloid fibrils by Aβ(1–40), Aβ(1–42), and α-synuclein [36]. On the other hand, tannic acid does not always show an inhibitory effect on amyloid fibrillation, and its modulation effect on amyloid fibrillation highly depends on the actual amyloid systems. For example, Boye-Harnasch et al. investigated the effect of tannic acid on the amyloid fibrillation of prion protein HET-s, and found that tannic acid behaved as an amyloid inducer and accelerated amyloid formation [37]. Khan et al. investigated the effect of tannic acid on the aggregation of concanavalin A and discovered that tannic acid could induce amyloid fibrillation [38]. Apparently, the modulation effect of tannic acid on amyloid fibrillation is quite complex and there is currently no unified picture about its role in amyloid fibrillation. To further look into the modulation effect of tannic acid on amyloid fibrillation, we performed a detailed atomic force microscopy (AFM) and fluorescence spectroscopy study of the effect of tannic acid on amyloid fibrillation using lysozyme as a model amyloid system. Interestingly, we discovered a novel modulation effect of tannic acid on amyloid fibrillation with the AFM technique. A hypothesis was proposed to interpret our AFM observation from the viewpoint of the surface-catalyzed nucleation mechanism. We further looked into the nature of molecular interaction between tannic acid and lysozyme using fluorescence quenching technique and revealed the role of hydrophobic interaction.

## 2. Results and Discussion

### 2.1. ThT Fluorescence Assay

**Figure 2:**
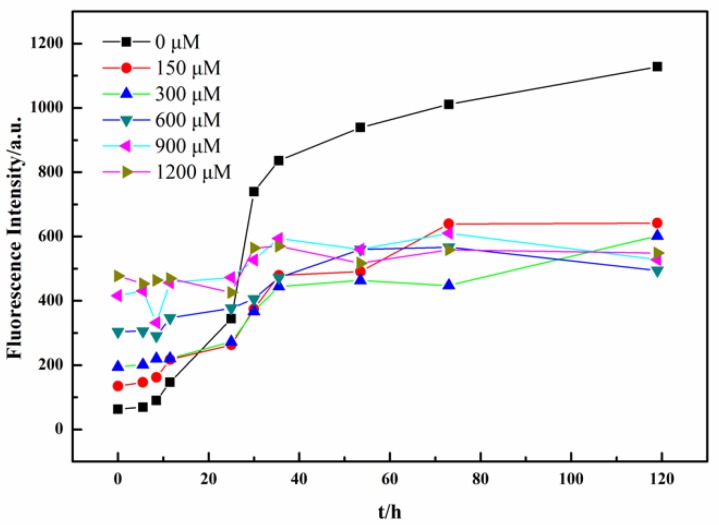
ThT fluorescence assay for the fibrillation kinetics of lysozyme.

We first used a ThT fluorescence assay to characterize the effect of tannic acid on the amyloid fibrillation process of lysozyme under heat and acidic conditions. Under the heat and acid conditions, the lysozyme undergoes partial hydrolysis at aspartic acid sites to produce highly amyloidogenic lysozyme fragments that form amyloid fibrils upon incubation [21]. ThT is a dye commonly used in the diagnostics of amyloid fibrils [6]. Its fluorescence intensity can be used to approximately quantify the amount of amyloid fibrils in amyloid kinetic studies. In general, ThT is specific to amyloid fibril, yet nonfibrillar structure may also cause ThT fluorescence intensity to increase [39]. In addition, multiple binding sites on the amyloid fibril could cause complications in the ThT assay; an alternative approach to better quantify amyloid fibrils is available in the literature [40]. Figure 2 shows the kinetic profile of lysozyme fibrillation in the absence and presence of tannic acid from a representative measurement. The kinetic curve in the absence of tannic acid displays a sigmoidal shape with a lag phase (i.e., the initial flat region), a growth phase (i.e., the slope region), and an equilibrium phase (i.e., the late flat region). The curve-fitting result is presented in Figure S1 in the Supplementary Material, which gives a lag-phase duration of about 7.7 h. As we can see, in the lag phase from t = 0 to ~7.7 h, there is no significant presence of amyloid fibril in the solution; in the growth phase from t = ~7.7 to 40 h, a significant number of fibrils are generated; after t = ~40 h, the system basically reaches an equilibrium. Such a sigmoidal curve is the kinetic feature of a typical amyloid fibrillation process. When tannic acids were added into the lysozyme amyloid system, significant changes of the kinetic curve were observed in Figure 2. First, at t = 0 h, when fibrillation had not even started, we already had some ThT fluorescence intensity, and the ThT intensity at t = 0 h also increased with tannic acid concentration. The observed ThT fluorescence intensity at t = 0 h in the presence of tannic acid should be due to the formation of nonfibrillar lysozyme aggregates that are ThT-active. Figure S2 shows that the ThT bound to the nonfibrillar aggregates displays an obvious fluorescence response. Such a fluorescence response is not due to the tannic acid, as tannic acid has no absorption at 450 nm, and does not produce fluorescence when excited at 450 nm, as indicated in Figure S2 as well. Second, the kinetic curves in the presence of tannic acid basically lost their well-defined sigmoidal shape, but still showed ThT intensity increase during incubation. In general, when an organic molecule behaves like an amyloid inducer, the sigmoidal kinetic curve shifts to the left as compared to the control; when an organic molecule behaves like an amyloid inhibitor, the sigmoidal kinetic curve shifts to the right when partial inhibition occurs, or becomes a flat line when complete inhibition occurs. Apparently, the effect of tannic acid on lysozyme amyloid fibrillation does not belong to either of the above two cases. Namely, tannic acid is neither a simple amyloid inducer nor a simple amyloid inhibitor. Rather, kinetic studies suggest a new modulation effect of tannic acid on amyloid fibrillation.

### 2.2. AFM Investigation

**Figure 3:**
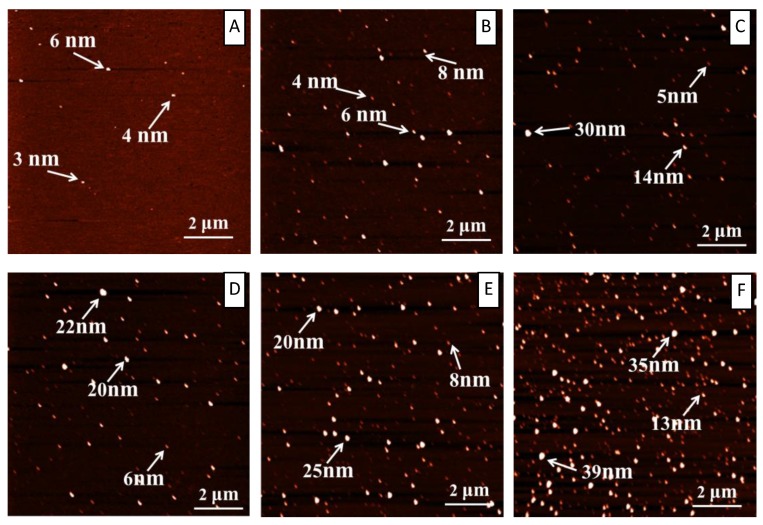
Atomic force microscopy (AFM) characterizations of lysozyme fibrillation under the influence of different concentrations of tannic acid at t = 0 h during incubation. (**A**) Control; (**B**) 150 µM; (**C**) 300 µM; (**D**) 600 µM; (**E**) 900 µM; (**F**) 1200 µM.

**Figure 4:**
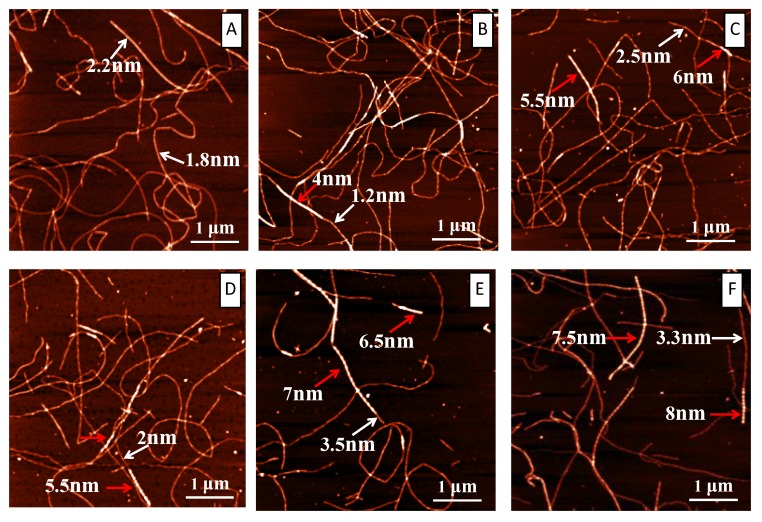
AFM characterizations of lysozyme fibrillation under the influence of different concentrations of tannic acid at the end of the incubation. (**A**) Control; (**B**) 150 µM; (**C**) 300 µM; (**D**) 600 µM; (**E**) 900 µM; (**F**) 1200 µM.

AFM is an excellent tool to visualize the modulation of tannic acid on the amyloid fibrillation of lysozyme at nanoscale. Two sets of AFM data were collected: one set was collected at the very beginning of the incubation, and the other was collected at the end of the incubation. Figure 3 shows the AFM images at t = 0 h for both the control system (A) and the system with tannic acids in different concentrations (B, C, D, E, and F). Figure 3A is the control and it is basically a clean mica surface. From Figure 3B–F, as the concentration of tannic acid increased from 150 to 1200 μM, we could see an increasing number of nonfibrillar aggregates on the mica surface. The size of these aggregates can reach tens of nanometers. These observations support that, at the beginning of the incubation, tannic acid instantly induced the formation of nonfibrillar aggregates by lysozyme. These amorphous aggregates bound ThT in a nonspecific manner and caused the initial ThT intensity to increase at t = 0 h, as shown in Figure 2 and Figure S2. Since more tannic acids lead to the formation of more amorphous aggregates, we thus observed that ThT intensity t = 0 h increased with the increase of tannic acid concentration. Figure 4 shows the AFM images at the end of the incubation for both the control system (A) and the system with tannic acids of different concentrations (B, C, D, E, and F). A glance of these images seems to support that there is no obvious effect of tannic acid on the amyloid fibrillation of lysozyme as in all of these images we can observe amyloid fibrils with similar morphologies. However, a close look of these images reveals that tannic acid promotes the formation of the amyloid fibril with a varying height along the fibril axis. As indicated by the red arrows in Figure 4B–F, in all of these images, in the presence of tannic acid we can observe that some amyloid fibrils possess some regions (i.e., the brighter regions) where the height of the fibril is substantially larger than that of the rest regions along the fibril. Though we also observed this type of fibril in Figure 4A in the absence of tannic acid, the occurrence of this type of fibril is apparently much more frequent in the presence of tannic acid. Moreover, the amyloid structures in the red-arrow-denoted regions seemed to grow on top of the original fibril and appeared to use the preformed fibril as a template. In addition, when we scanned the AFM image at the end of the incubation, in the presence of tannic acid we always found regions on the mica surface that contained amorphous aggregates (as shown in Figure S3), indicating the coexistence of nonfibrillar aggregates with fibrillar aggregates in our incubation solution. In summary, AFM results suggest that tannic acid promotes the formation of an amyloid fibril with an interesting mixed morphology. Such a mixed morphology features with the existence of areas of thickening alternating with areas of normal height along fibril axis. This is a novel modulation effect of tannic acid on amyloid fibrillation that has never been reported.

**Figure 5:**
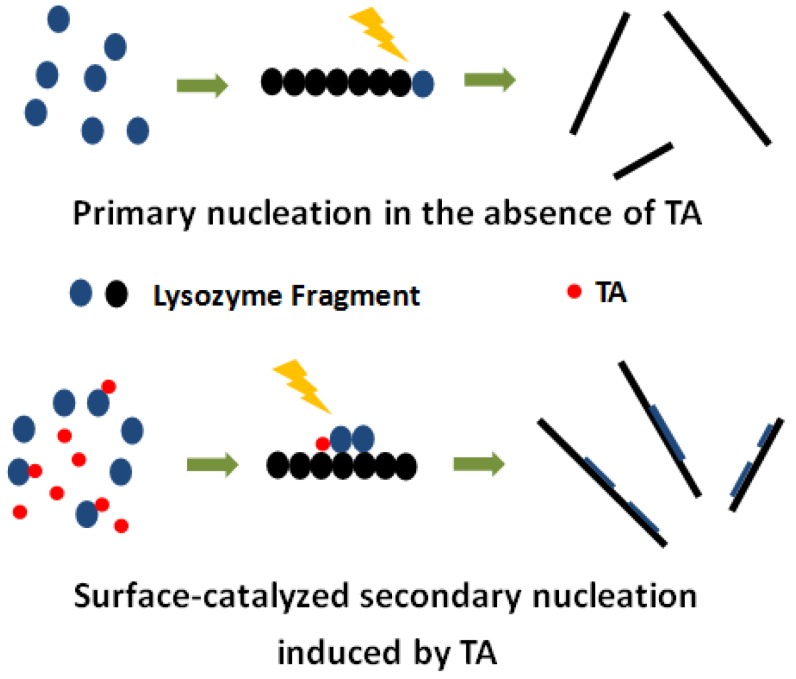
Schematic illustration of tannic acid-induced surface-catalyzed secondary nucleation.

In the following, we propose a hypothesis to interpret the observed modulation behavior of tannic acid on amyloid fibrillation based on surface-catalyzed nucleation theory. As we mentioned above, in surface-catalyzed nucleation, monomers use the surface of a preformed fibril as a type of template to nucleate. Surface-catalyzed nucleation could have significant impact on fibril morphology. As demonstrated in the works by Lashuel and Dietler, surface-catalyzed nucleation can lead to two types of morphology changes, fibril branching and fibril thickening [14]. In the first case, amyloidogenic peptide monomer uses some points at the surface of the preformed fibril as nucleation sites to let new fibrils “grow out of the preformed fibril”, leading to the formation of branched amyloid fibril. In the second case, the amyloidogenic peptide monomer uses some points at the surface of the preformed fibril as nucleation sites to let new fibrils “grow along the preformed fibril”. This allows the formation of thicker regions along an amyloid fibril. The latter case is exactly what we observed in Figure 4, suggesting a tannic acid-induced surface-catalyzed secondary nucleation mechanism. A likely scenario could be that the tannic acid interacts with the lysozyme to form a complex. With the aid of tannic acid, such a complex could be more easily anchored at the surface of an amyloid fibril to form a nucleation site to initiate the surface-catalyzed secondary nucleation process. In Figure 5, we schematically illustrated our hypothesis on the effect of tannic acid on amyloid fibrillation. In the absence of tannic acid, amyloid fibril with basically uniform height is formed; in the presence of tannic acid, amyloid fibril with alternating thickness is formed. It should be pointed out that, even in the absence of tannic acid, we can still occasionally observe an amyloid fibril with alternating thickness. Yet, with tannic acid, the possibility of the formation of an amyloid fibril with alternating thickness is significantly increased. This apparently supports the argument that tannic acids could induce surface-catalyzed secondary nucleation in amyloid fibrillation. To have such a modulation effect, there must be a type of intermolecular interaction between tannic acid and lysozyme. In the following, we use well-established fluorescent techniques, such as fluorescent quenching, to address this issue.

### 2.3. Fluorescence Quenching Investigation at 323, 328, and 333 K.

We used fluorescence quenching to study the interaction between lysozyme and tannic acid. The intrinsic fluorescence of a protein is sensitive to the structural change of a protein and the intermolecular interaction between a protein and a ligand and can thus serve as a spectroscopic probe. The intrinsic fluorescence of a protein arises from three fluorescent amino acid residues in protein, namely, phenylalanine, tyrosine, and tryptophan. Although tyrosine has a quantum yield similar to tryptophan, the indole group of tryptophan is considered the dominant source of UV absorbance at 280 nm and emission at 350 nm in proteins. Therefore, in general, tryptophan is the dominant source of fluorescent emission at 350 nm when the protein is excited at 280 nm [41]. Lysozyme is a relatively small monomeric low-molecular-weight globular protein with 129 amino acid residues (MW = 14,307), including six tryptophans and three tyrosines. In Figure S4, we showed the fluorescence spectra of lysozyme excited at 280 and 297 nm, respectively. As we can see, the peak positions of the two spectra are the same, suggesting that the dominant fluorescence contribution in the lysozyme system is from tryptophan and is not affected by different excitation wavelengths.

#### 2.3.1. Fluorescence Quenching Spectra of the Lysozyme–Tannic Acid System

**Figure 6:**
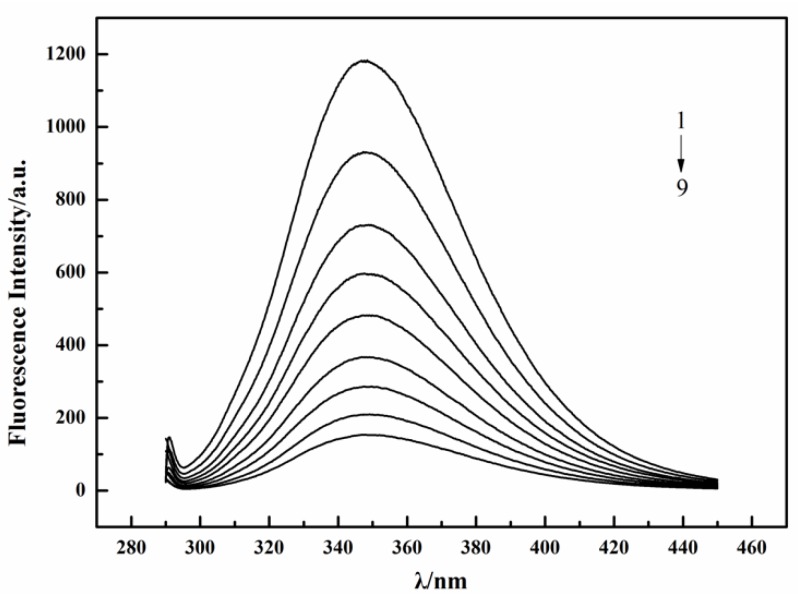
Fluorescence spectra of lysozyme quenched by tannic acid at 333 K. C_Lysozyme_ = 3.6 × 10^−6^ mol/L; C_Tannic acid_ (1–9) = (0, 1.8, 3.6, 5.4, 7.2, 9.0, 10.8, 14.4, 18.0) × 10^−6^ mol/L.

Figure 6 showed a series of fluorescence spectra of lysozyme excited at 280 nm upon addition of different concentrations of tannic acid at 333 K. The experiments were performed at the fibrillation temperature of 333 K (i.e., 60 °C). These spectra have fluorescence emission maxima at ~348 nm. As we can see, fluorescence intensity decreased with the increase of tannic acid concentrations, and there was no shift of emission wavelength among these spectra. This observation suggests that the intrinsic fluorescence of lysozyme was quenched by tannic acid and there was an interaction between lysozyme and tannic acid.

To quantitatively analyze the fluorescence quenching data in Figure 6, we first performed a correction on fluorescence intensity by taking inner filter effects (IFE) into account. In general, IFE refers to the absorption of light by other compounds in the solution at the excitation or emission wavelength, which results in a nonlinear decrease in fluorescence intensity with the increase of the quencher’s concentration [42]. In order to eliminate the interference of IFE with the result, the following equation was adopted to correct the fluorescence intensity of lysozyme:

(1) $$F_{cor}=F_{obs}\times e^{\left(A_{ex}+A_{em}\right)/2}$$

where *F_cor_* and *F_obs_* are the corrected and observed fluorescence intensity of lysozyme, and *A_ex_* and *A_em_* are the absorbance of tannic acid at the excitation and emission wavelengths, respectively [42]. Equation (1) can approximately correct IFE, and such an equation has been widely adopted in the literature [43,44,45]. However, to obtain more accurate results, a newer approach proposed by Fonin et al. can be adopted [46]. Using Equation (1), we corrected the fluorescence intensities in our work.

**Figure 7:**
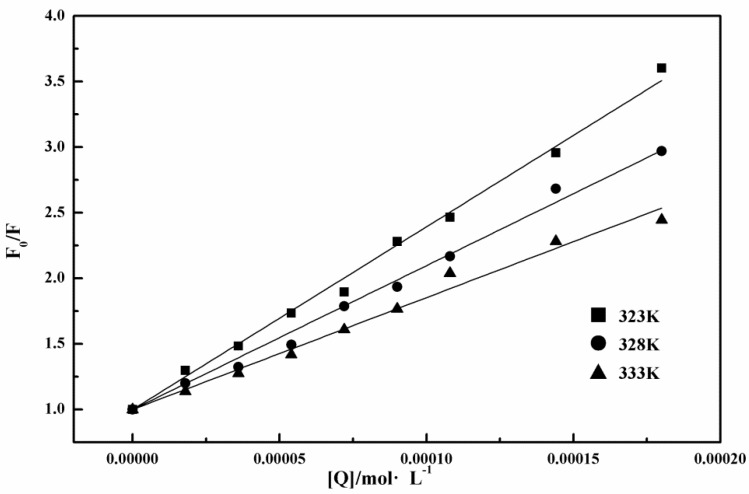
Stern–Volmer plots for lysozyme quenched by tannic acid at 323, 328, and 333 K.

The quenching mechanism is usually classified into static quenching or dynamic quenching. Dynamic and static quenching can be distinguished by their different temperature-dependence behaviors. For static quenching, quenching rate constants decrease with rising temperature; for dynamic quenching, quenching rate constants increase with rising temperature [42]. In order to identify the actual quenching mechanism, a temperature-dependent fluorescence quenching study was performed and the data were analyzed using the Stern–Volmer equation (Equation (2)), which gives the quenching rate constant [42]:

(2) $$F_{0}/F=1+K_{q}\tau_{0}\left[Q\right]=1+K_{sv}\left[Q\right]$$

where *F*_0_ is the fluorescence intensity of lysozyme; *F* is the fluorescence intensity of lysozyme in the presence of tannic acid; *K_q_* is the rate constant of the bimolecular quenching process; *τ*_0_ is the average life time of fluorescence without the quencher; *K_sv_* is the Stern–Volmer quenching constant; [*Q*] is the concentration of tannic acid. Previous studies showed that the actual fluorescence lifetime of tryptophan depended on the actual environment and could vary from 1 to 9 ns [41,47] (in this work, 9 ns was adopted). The dynamic quenching mainly depends on the molecular diffusion. The higher the temperature is, the larger the diffusion coefficient is, and the quenching constant thus increases with the increase of temperature. Static quenching give an opposite trend: the higher the temperature is, the lower the stability of ground-state complexes, and the smaller the quenching constant is. As shown in Figure 7, the linear fitting of *F*_0_*/F* to [*Q*] gives three slopes at three different temperatures. The obtained values of *K_sv_* and the derived *K_q_* are also listed in Table 1. As we can see in Figure 7 and Table 1, the slope (i.e., *K_sv_*) decreased with the increase of temperature, indicating that quenching constant *K_sv_* decreases with the increase of temperature, suggesting that the quenching of tannic acid for lysozyme is static quenching. Moreover, quenching-rate constant *K_q_* was larger than the maximum diffusion collision quenching constant of macromolecules which is 2.0 × 10^10^ L·mol^−1^·S^−1^ [42,43]. This observation further indicates that the quenching mechanism of tannic acid to lysozyme is static quenching.

#### 2.3.2. Association Constants and Number of Binding Sites

The binding parameters between lysozyme and tannic acid can be calculated using the following Equation (3) [42,44,48]:

(3) $$\mathrm{lg}[\left(F_{0}-F\right)/F]=\mathrm{lg}K_{A}+n\mathrm{lg}\left[Q\right]$$

where *F*_0_ is the fluorescence intensity of lysozyme; *F* is the fluorescence intensity of lysozyme in the presence of tannic acid; [*Q*] is the concentration of tannic acid; *K_A_* is the association constant; and *n* is the number of substantive binding sites. The curves of lg[(*F*_0_ − *F*)/*F*] versus lg[*Q*] were plotted according to Equation (3), and the values of *K_A_* and *n* are obtained from the intercept and slope of the curves, respectively and shown in Table 2. The values of *n* were approximately equal to 1 at different temperatures, indicating there is one binding site of tannic acid to lysozyme. The binding constants of tannic acid and lysozyme at different temperatures were in the order of about 10^4^, suggesting that tannic acid and lysozyme are bound to some extent.

#### 2.3.3. Determination of Binding Forces

In general, the binding forces between quencher and protein are noncovalent in nature and include the following interaction forces: hydrogen bonding, electrostatic interaction, van der Waals force, and hydrophobic force. Ross and Subramanian summed up the thermodynamic laws from thermodynamic parameters, including enthalpy change (Δ*H*), entropy change (Δ*S*), and free energy change (Δ*G*), to determine the binding types [49]. If Δ*H* ˂ 0 and Δ*S* ˂ 0, the main forces are van der Waals and hydrogen-bond interactions; if Δ*H* ˂ 0 and Δ*S* > 0, the electrostatic effect is dominant; if Δ*H* > 0 and Δ*S* > 0, the main force is hydrophobic interaction. The thermodynamic data calculated according to Equations (4)–(6) are given in Table 3, where Δ*H* is considered constant, as the change of temperature is small. The corresponding Van’t Hoff plot is presented in Figure S5.

(4) $$\ln K=-\frac{\Delta H}{RT}+C$$

(5) ΔG=−RTlnK

(6) ΔS=(ΔH−ΔG)/T

In our case, Δ*G* ˂ 0, indicating the interaction between lysozyme and tannic acid was spontaneous; Δ*H* > 0 and Δ*S* > 0, indicating that the main interaction between tannic acid and lysozyme is a hydrophobic interaction in the temperature range of 323 to 333 K.

#### 2.2.4. Binding Distances

**Figure 8:**
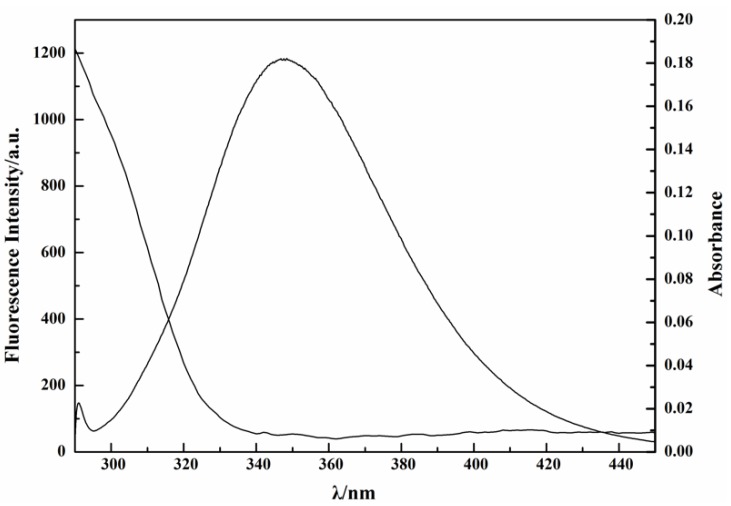
Overlap of fluorescence emission spectrum of lysozyme (1) with the absorption spectrum of tannic acid (2) at T = 333 K.

Binding distance was studied according to Föster’s nonradioactive resonance energy-transfer theory [24]. Nonradiative energy transfer occurs when the two compounds meet the following three preconditions: (1) the donor is a fluorophore; (2) the fluorescence-emission spectrum of the donor is sufficiently overlapped with the absorption spectrum of the acceptor; (3) the donor is close enough to the acceptor, and the maximum distance is less than 7 nm. The overlap of the fluorescence-emission spectrum of lysozyme with the absorption spectrum of tannic acid is shown in Figure 8, which shows sufficient overlap between these two spectra. According to Föster’s theory, the relationship between energy-transfer efficiency *E* and distance *r* is as follows [43,50]:

(7) $$E=1-F/F_{0}=R_{0}^{6}/(R_{0}^{6}+r^{6})$$

where *R*_0_ is the critical distance that is the distance between tannic acid and lysozyme when transfer efficiency is 50% and given by Equation (8), and *r* is the distance between tannic acid and lysozyme.

(8) $$R_{0}^{6}=8.78\times 10^{-25}K^{2}\Phi N^{-4}J$$

In Equation (8), *K*^2^ is the dipole space orientation factor; *N* is the refractive index of the medium; *Φ* is the fluorescence quantum yield of donor; it was reported that, for lysozyme, *K*^2^ = 2/3; *N* is the water refraction index, which is 1.336; and *Ф* = 0.15 [50]. *J* is the overlap integral between the fluorescence spectra of the donor and the absorption spectrum of the acceptor as described in Equation (9):

(9) $$J=\sum F\left(\lambda \right)\varepsilon \left(\lambda \right)\lambda^{4}\Delta \lambda /\sum F\left(\lambda \right)\Delta \lambda$$

where *F(λ)* and *ε(λ)* are the fluorescence intensity of lysozyme at the wavelength of λ and the molar absorptivity of tannic acid at λ, respectively. The *E*, *J*, *R*_0_, and *r* obtained from the formula are shown in Table 4. The distance of donor to acceptor was found to be *r* < 7 nm, indicating the existence of nonradiative energy transfer between tannic acid and lysozyme. Thus, nonradiative energy transfer is one of the causes of fluorescence quenching. Table 4 also shows that, with the increase of temperature, energy-transfer efficiency *E* decreases and binding distance increases; on the other hand, critical distance *R*_0_ is almost unchanged, indicating the critical distance between tannic acid and lysozyme is not affected by temperature.

#### 2.2.5. Fluorescence Quenching Investigation at 298, 303, and 308 K.

Fluorescence investigation performed in the temperature range of 323 ~ 333 K indicated that the interaction between lysozyme and tannic acid is hydrophobic-interaction-driven. As we know, tannic acid contains a large amount of hydroxyl groups that can form hydrogen bonding with its surroundings. One may be curious why the interaction between lysozyme and tannic acid is not hydrogen-bonding-driven. The following experiment performed in a lower temperature range might provide some insight. We performed the fluorescence quenching investigation in the temperature range of 298 ~ 308 K. The corresponding results are included in Figures S6 and S7, Tables S1–S3, and Table 5 (the corresponding Van’ t Hoff plot was presented in Figure S8.). Among these results, the most interesting is shown in Table 5. In the 298 to 308 K range, we found that Δ*H* ˂ 0 and Δ*S* ˂ 0. This indicates that the main interaction between tannic acid and lysozyme is a hydrogen bond and van der Waals force at this temperature range. As we mentioned above, in the 323 to 333 K range, Δ*H* > 0 and Δ*S* > 0. This indicates that the main interaction between tannic acid and lysozyme is hydrophobic interaction at this temperature range. Clearly, the nature of the interaction between tannic acid and lysozyme is temperature-dependent. We believe the observed temperature-dependent phenomenon is related to the temperature-dependent stability of hydrogen bonds. At higher temperatures, a hydrogen bond is weakened, and tannic acid thus tends to mainly use its hydrophobic aromatic rings to interact with lysozyme; at a lower temperature, a hydrogen bond is more stable and tannic acid thus tends to mainly use its large amount of hydroxyl groups to interact with lysozyme. In addition, higher temperature favors the exposure of hydrophobic residues of lysozyme to make them more accessible to tannic acid. Our lysozyme fibrillation was performed in the high temperature range, so the interaction between lysozyme and tannic acid during amyloid fibrillation is driven by hydrophobic interaction.

Polyphenols like tannic acid and other related compounds have both aromatic and polar functional groups, and their interactions with proteins could involve multiple intermolecular forces, including hydrogen bonding, electrostatic interaction, van der Waals force, and hydrophobic force. These forces could be present simultaneously. For example, Mohammadi et al. studied the inhibitory activity of some curcuminoids on the amyloid fibrillation of lysozyme [50]. On one hand, their molecular docking results indicate that the interaction between curcuminoid and lysozyme involves both hydrophobic force and hydrogen bonding. On the other hand, their thermodynamic analysis on the fluorescence quenching data indicated that a hydrogen-bonding interaction was the dominant interaction between curcuminoid and lysozyme according to Ross’s thermodynamic law [50]. Our study further shows that the dominant interaction between polyphenols (i.e., tannic acid in this work) and lysozyme could be temperature-dependent, namely, different interaction forces play dominant roles in different temperature ranges.

Tannic acid in this work apparently has two effects on the aggregation of the lysozyme. One is the novel modulation effect of tannic acid on lysozyme amyloid fibrillation through surface-catalyzed secondary nucleation; the other is tannic acid-induced formation of amorphous aggregates. The fact that tannic acid can have two different effects on the same protein is reasonable. In amyloid formation and protein aggregation, polymorphism is a common phenomenon [30,51]. In other words, it is very common to observe that the same protein can produce different types of aggregates during incubation. In our case, under the influence of tannic acid, lysozyme produced two different types of aggregates, amorphous aggregate and fibrillar aggregate. We further hypothesize that the modulation effects of tannic acid should be relevant to its molecular structure. Tannic acid possesses a relatively large amount of hydroxyl and aromatic groups. This makes it easy to have more interaction sites with its surroundings. It could serve as a “bridging” molecule to bring more lysozyme monomers together to form an amorphous aggregate or anchor lysozyme–tannic acid complexes onto a pre-existing amyloid fibril surface to initiate the surface-catalyzed secondary nucleation process.

## 3. Materials and Methods

### 3.1. Materials

Hen egg white lysozyme (L6876) was obtained from Sigma-Aldrich (Saint Louis, MO, USA) and used directly. Sodium chloride (NaCl) with >99% purity was purchased from Sigma Aldrich (Saint Louis, MO, USA). Tannic acid in analytical grade was purchased from Aladdin (Shanghai, China). Hydrochloride acid (HCl) in analytical grade with a purity of 36–38% was purchased from Kemiou Chemical Reagent (Tianjin, China). Thioflavin T (ThT) was purchased from Acros (Geel, Belgium). No further purification was performed on all the chemicals. Deionized water with a resistivity of 18.2 MΩ·cm was obtained from a Millipore system (Billerica, MA, USA). Mica (muscovite) was obtained from a local vendor.

### 3.2. Amyloid Fibrillation

Lysozyme was first dissolved in the HCl solution with pH = 2 and 10 mg/mL concentration in glass vial. The solution was incubated at 333 K in a thermos-shaker heater from 24 h. This step was to activate lysozyme through partial hydrolysis at aspartic acid sites to produce amyloidogenic lysozyme fragments [21]. This approach had been used by others and by us as an easy way to prepare lysozyme amyloid fibrils [21,29,30]. The hydrolyzed lysozyme solution was then used in the fibrillation study as well as the fluorescence study. To make amyloid fibrils, the hydrolyzed lysozyme solution was aliquoted into new glass vials to make the incubation solutions for amyloid fibrillation in the absence or in the presence of tannic acid at pH = 2 and 333 K. The incubation solution contained 5 mg/mL lysozyme and 140 mM NaCl. The following concentrations of tannic acid were used: 150, 300, 600, 900, and 1200 μM, respectively.

### 3.3. AFM

All AFM images were taken on dried samples in air with an NT-MDT Solver P47 scanning probe microscope (Zelenograd, Russia) in tapping mode. A 100 μm × 100 μm scanner was used throughout the AFM experiment. The silicon cantilever was purchased from NT-MDT. The cantilever has a resonance frequency of ∼100 kHz and a nominal force constant of ∼3 N/m. The AFM measurement samples were prepared using the following protocol: a 10 µL sample was taken out of the incubation vial and immediately diluted 400 times with deionized water. 10 µL of the diluted solution was dropped onto a piece of freshly cleaved mica. After 3 min waiting time, the sample solution was rinsed off with deionized water. The mica sample was further dried in an oven at 37 °C for at least 2 h and the dried mica surface was subjected to AFM measurement. AFM images were analyzed by NT-MDT software, NOVA.

### 3.4. ThT Fluorescence Assay

ThT fluorescence assay was performed with a Hitachi F-7000 fluorescence spectrophotometer (Tokyo, Japan). The following acquisition parameters are used: excitation (ex) wavelength, 450 nm; ex slit width, 5 nm; emission (em) wavelength, 486 nm; em slit width, 10 nm; photomultiplier (PMT) voltage, 700 V; scan speed, 60,000 nm/min. The concentration of ThT solution is 10 μM. The buffer is 20 mM phosphate buffer at pH = 7.4. The assay was performed ex situ. During fibrillation, aliquots of incubation solutions were taken out of the incubation vial at different time points and were subjected to the assay immediately. For each measurement, 10 μL of incubation solution was added into 1 mL of ThT solution in a 1.0 cm quartz cuvette. The measurements were performed under ambient conditions.

### 3.5. UV-Vis Measurement

UV–vis spectra were recorded on an Implen Nanophotometer spectrophotometer (München, Germany). The spectra of the solutions were scanned in the wavelength region of 200 to 600 nm using a 1 cm quartz cuvette.

### 3.6. Fluorescence Quenching Measurements

Fluorescence measurements were performed with a Hitachi F-7000 fluorescence spectrophotometer (Tokyo, Japan). The acquisition parameters for fluorescence quenching were: ex, 280 nm; ex slit width, 5 nm; em, 290–450 nm; em slit width, 10 nm; PMT, 700 V; scan speed, 1200 nm/min. The concentration of lysozyme was 3.6 µM.

## 4. Conclusions

In summary, we investigated the modulation effect of tannic acid on amyloid fibrillation by hen egg white lysozyme using the AFM and fluorescence spectroscopy techniques. We discovered that tannic acid could promote surface-catalyzed secondary nucleation during lysozyme fibrillation. This novel modulation effect of tannic acid leads to a substantial morphology change in amyloid fibrils. The amyloid fibril under the influence of tannic acid features with the existence of areas of thickening alternating with areas of normal height. We further performed fluorescence quenching studies to investigate the intermolecular interaction between tannic acid and lysozyme, and found that lysozyme and tannic acid interact with each other mainly through hydrophobic interaction at higher temperature. We also discussed why the interaction between tannic acid and lysozyme is not hydrogen-bonding-driven, though tannic acid contains a significant amount of hydroxyl groups. Our work provides new insight into the effect of tannic acid and related polyphenols on amyloid fibrillation.

## Figures and Tables

**Table 1:** Stern–Volmer parameters of a lysozyme–tannic acid system at 323, 328, and 333 K.

T/K	*K_sv_*/(L·mol^−1^)	*K_q_*/(L·mol^−1^·s^−1)^	r *
323	(1.30 ± 0.10) × 10^4^	(1.44 ± 0.11) × 10^12^	0.9948 ± 0.0024
328	(1.01 ± 0.09) × 10^4^	(1.12 ± 0.10) × 10^12^	0.9926 ± 0.0030
333	（(0.97 ± 0.11) × 10^4^	(1.08 ± 0.12) × 10^12^	0.9915 ± 0.0044

* r is correlation coefficient.

**Table 2:** Binding parameters of a lysozyme–tannic acid system at 323, 328, and 333 K.

T/K	*K_A_*/(L·mol^−1^)	*n*	r *
323	(6.83 ± 4.09) × 10^3^	0.91 ± 0.09	0.9901 ± 0.0051
328	(2.16 ± 1.59) × 10^4^	1.07 ± 0.09	0.9943 ± 0.0016
333	(4.24 ± 4.02) × 10^4^	1.13 ± 0.09	0.9968 ± 0.0020

* r is correlation coefficient.

**Table 3:** Thermodynamic parameters of the lysozyme–tannic acid system at 323, 328, and 333 K.

T/K	Δ*H*/(kJ·mol^−1^)	r *	Δ*G*/(kJ·mol^−1^)	Δ*S*/[J·(mol·K^−1^)^−1^]
323	139.38	0.9901	−21.88	541.13
328			−25.14	542.99
333			−27.28	541.11

* r is correlation coefficient.

**Table 4:** Parameters of *E*, *J*, *R*_0_, *r* of the lysozyme–tannic acid system at 323, 328 and 333 K.

*T*/K	*E*/%	*J*/(cm^3^·L·mol^−1^)	*R*_0_/nm	*r*/nm
323	32.45 ± 0.35	(5.95 ± 0.04) × 10^−15^	2.34 ± 2.48 × 10^−3^	2.64 ± 0.01
328	23.51 ± 3.88	(6.00 ± 0.04) × 10^−15^	2.34 ± 2.78 × 10^−3^	2.86 ± 0.11
333	22.95 ± 1.55	(6.15 ± 0.26) × 10^−15^	2.34 ± 1.63 × 10^−2^	2.88 ± 0.02

**Table 5:** Thermodynamic parameters of the lysozyme–tannic acid system at 298, 303, and 308 K.

T/K	Δ*H*/(kJ·mol^−1^)	r *	Δ*G*/(kJ·mol^−1^)	Δ*S*/[J·(mol·K^−1^)^−1^]
298	−97.28	0.9999	−28.92	−229.42
303			−27.80	−229.30
308			−26.62	−229.42

* r is correlation coefficient.

## Supplementary Materials

Supplementary materials can be found at http://www.mdpi.com/1422-0067/19/12/4009/s1.
