# Supplementary material for: Tannic Acid-Induced Surface-Catalyzed Secondary Nucleation during the Amyloid Fibrillation of Hen Egg-White Lysozyme
Source: Int J Mol Sci. 2018 Dec 12;19(12):4009. doi: 10.3390/ijms19124009 (PMC6320987; doi:10.3390/ijms19124009)
Supplement: Supplementary file 1 [file ijms-19-04009-s001.pdf]

Tannic Acid-Induced Surface-Catalyzed Secondary Nucleation during the Amyloid Fibrillation of Hen Egg White Lysozyme

Jing Tian, Yang Yu, Yao Wang, Haoyi Li, Lujuan Yang, Baoan Du* and Gang Ma*

Key Laboratory of Medicinal Chemistry and Molecular Diagnosis of Ministry of Education, Key Laboratory of Analytical Science and Technology of Hebei Province, College of Chemistry and Environmental Science, Hebei University, Baoding 071002, China; 1270162026@qq.com (J.T.); 1523785107@qq.com (Y.Y.); 2051826190@qq.com (Y.W.); haoyili.zh@gmail.com (H.L.); 1960214805@qq.com (L.Y.)

* Correspondence: baoandu@sina.com (B.D.); gangma@hbu.edu.cn (G.M.); Tel.: +86-312-5077393 (G.M.)

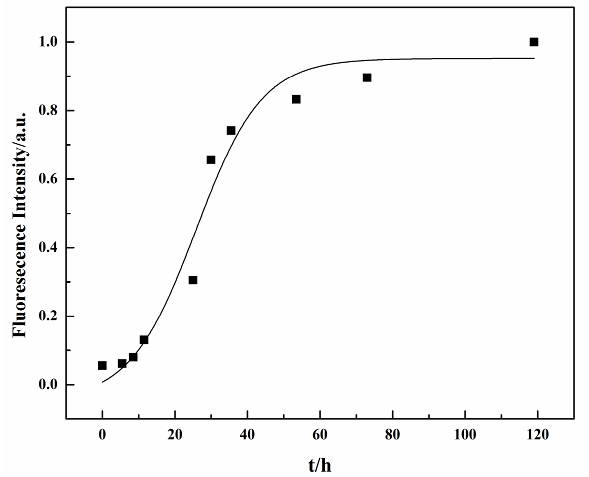

Figure S1. ThT fluorescence assay for the fibrillation kinetics of lysozyme with empirical fit using the equation of $F = F_0 + 1/\left(1+\exp\left[r_{max}\left(\tau_{1/2}-t\right)\right]\right)$, where $\tau_{1/2}$ is the time for half completion of aggregation, r_{max} is the maximum growth rate, and $\tau_{1/2}$-2/r_{max} is the lag phase time duration [1].

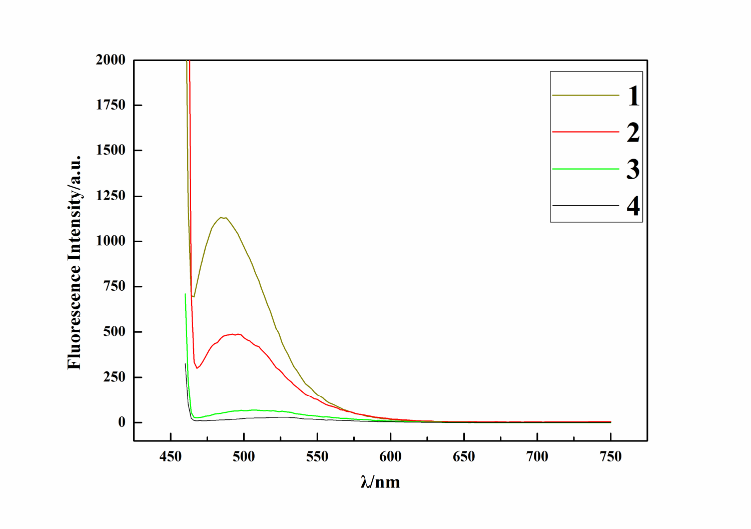

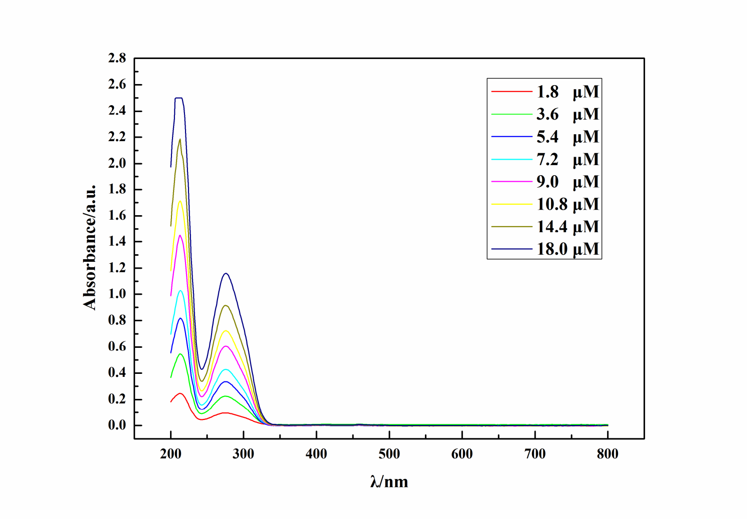

Figure S2. (**Top**) Fluorescence spectra of ThT bound to amyloid fibril. C_{ThT} =10μM; Incubation condition: $C_{lysozyme}$=5mg/mL; t=120h (1); ThT bound to non-fibrillar aggregates C_{ThT} =10μM; Incubation condition: $C_{lysozyme}$=5mg/mL; $C_{Tannic\ acid}$=1200μM; t=0h (2); the control (i.e., ThT + tannic acid), C_{ThT} =10μM (3); and the control (tannic acid alone), $C_{Tannic\ acid}$=1200μM (4). (**Bottom**) Absorption spectra of tannic acid under different concentrations.

Note: The ThT assay was performed ex situ. For each ThT assay, only 10 μl of the test solution was added in to 1 mL of ThT solution. So in the test solution in ThT assay, the concentration of ThT is 10 μM; the concentration of lysozyme is 0.05 mg/ml, and the concentration of tannic acid is only 12 μM.

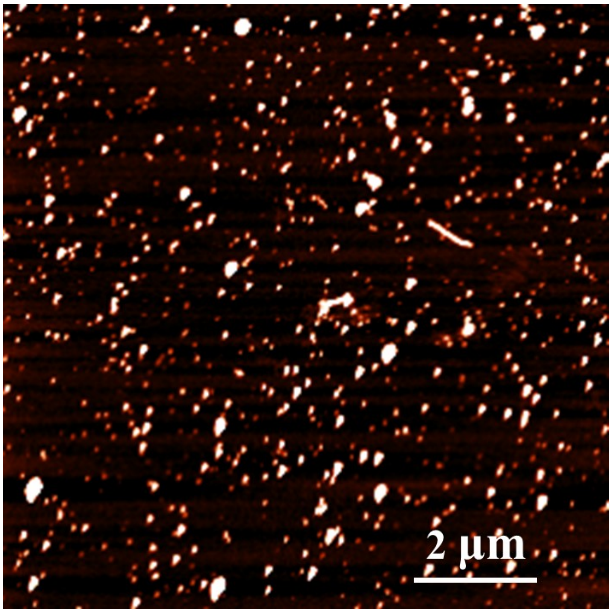

Figure S3. AFM evidence for the presence of the non-fibrillar aggregates at the end of incubation in the presence of tannic acid (i.e., t=137h). $C_{lysozyme}$=5mg/mL; $C_{Tannic\ acid}$=1200μM. This image was from the same mica surface for Figure 4F.

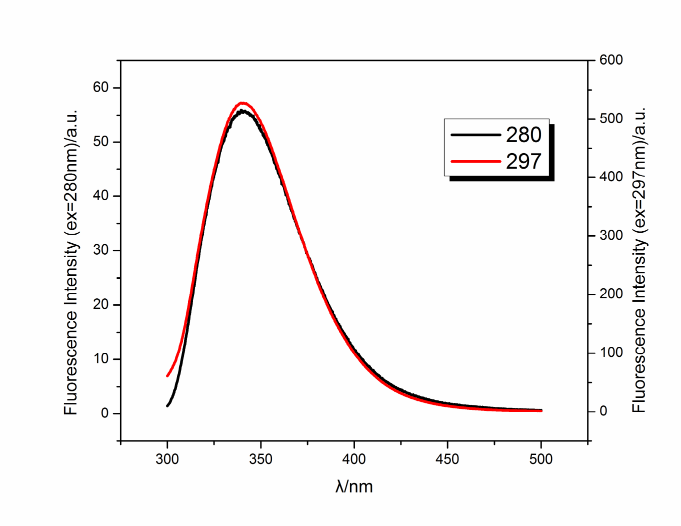

Figure S4. Fluorescence spectra of lysozyme at 5mg/mL excited with 280 nm and 297 nm.

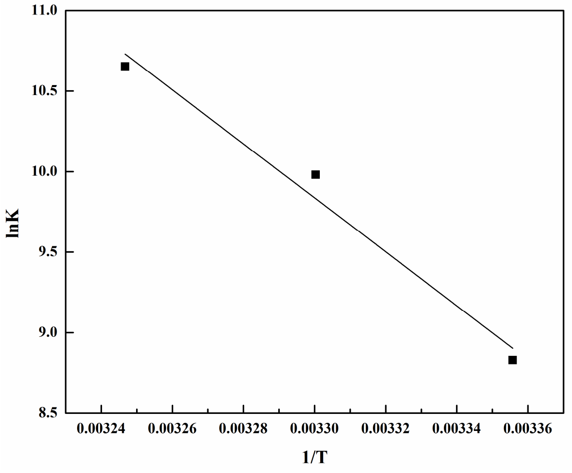

Figure S5. Van 't Hoff plot for lysozyme quenched by tannic acid at 323 K, 328 K and 333 K.

$C_{lysozyme}$=3.6×10^{-6}mol/L; $C_{Tannic\ acid}$ (1–9) = (0, 1.8, 3.6, 5.4, 7.2, 9.0, 10.8, 14.4, 18.0)×10^{-6}mol/L

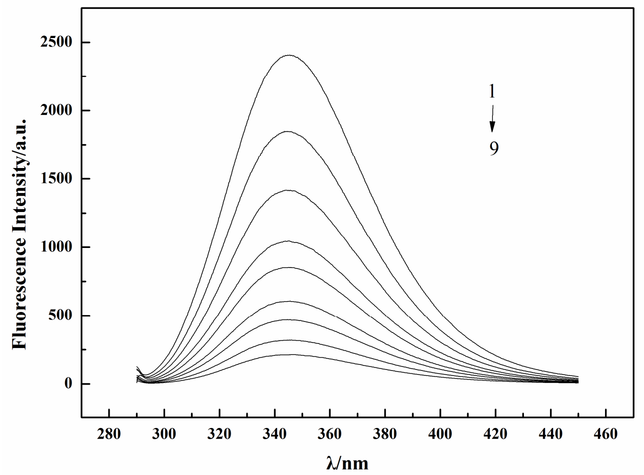

Figure S6. Fluorescence spectra of lysozyme quenched by tannic acid at 298 K.

$C_{lysozyme}$=3.6×10^{-6}mol/L; $C_{Tannic\ acid}$ (1–9) = (0, 1.8, 3.6, 5.4, 7.2, 9.0, 10.8, 14.4, 18.0)×10^{-6}mol/L

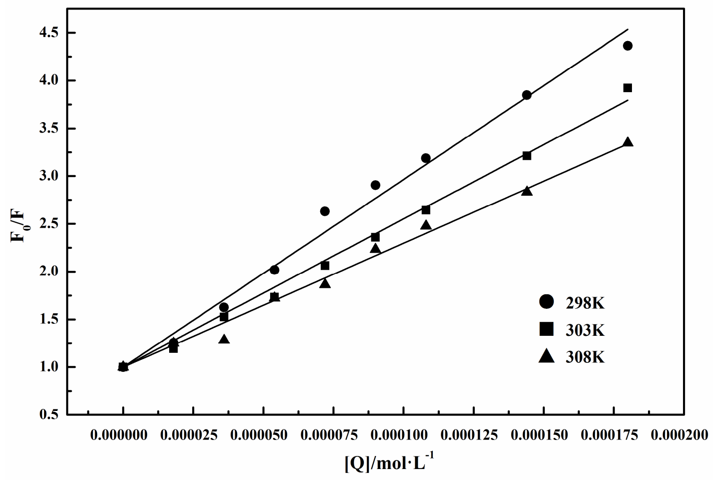

Figure S7. Stern-Volmer plots for lysozyme quenched by tannic acid at 298 K, 303 K and 308 K.

Table S1. The Stern-Volmer parameters of lysozyme-tannic acid system at 298 K, 303 K and 308 K.

T/K	$K_{sv}/(L\cdot mol^{-1})$	$K_q/(L\cdot mol^{-1}\cdot s^{-1})$	r*
298	$(1.65\pm0.28)\times10^4$	$(1.83\pm0.31)\times10^{12}$	0.9897±0.0081
303	$(1.47\pm0.08)\times10^4$	$(1.63\pm0.09)\times10^{12}$	0.9944±0.0030
308	$(1.34\pm0.13)\times10^4$	$(1.49\pm0.14)\times10^{12}$	0.9917±0.0039

*r is correlation coefficient.

Table S2. Binding parameters of lysozyme-tannic acid system at 298 K, 303 K and 308 K.

T/K	$K_A/(L\cdot mol^{-1})$	*n*	r
298	$(1.17\pm0.86)\times10^5$	1.20±0.08	0.9931±0.0051
303	$(6.20\pm4.54)\times10^4$	1.13±0.10	0.9936±0.0036
308	$(3.27\pm1.03)\times10^4$	1.10±0.03	0.9866±0.0114

Table S3. Parameters of *E*, *J*, R_0, *r* of the lysozyme-tannic acid system at 298 K, 303 K and 308 K.

T/K	*E*/%	$J/(cm^3\cdot L\cdot mol^{-1})$	R_0/nm	*r*/nm
298	32.43±8.52	$(5.96\pm0.01)\times10^{-15}$	$2.34\pm3.27\times10^{-4}$	2.65±0.17
303	31.48±3.02	$(5.99\pm0.04)\times10^{-15}$	$2.34\pm2.34\times10^{-3}$	2.67±0.06
308	26.61±4.28	$(5.96\pm0.06)\times10^{-15}$	$2.34\pm4.18\times10^{-3}$	2.78±0.11

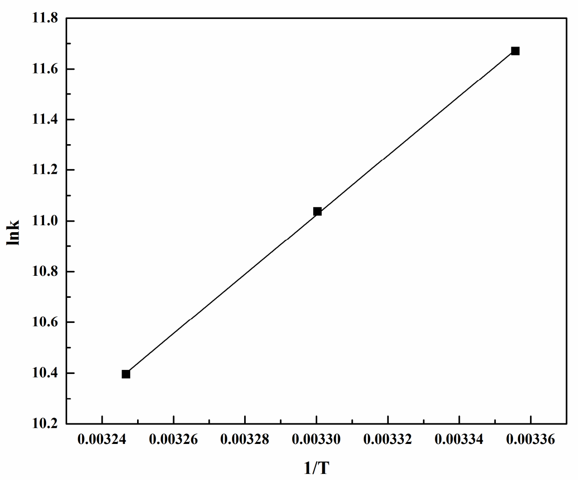

Figure S8. Van 't Hoff plot for lysozyme quenched by tannic acid at 298 K, 303 K and 308 K.

$C_{lysozyme}$=3.6×10^{-6}mol/L; $C_{Tannic\ acid}$ (1–9) = (0, 1.8, 3.6, 5.4, 7.2, 9.0, 10.8, 14.4, 18.0)×10^{-6}mol/L

1. Abelein, A.; Jarvet, J.; Barth, A.; Graslund, A.; Danielsson, J. Ionic strength modulation of the free energy landscape of a beta(40) peptide fibril formation. *J. Am. Chem. Soc.* **2016,** *138,* 6893-6902, 10.1021/jacs.6b04511
